# Supplementary material for: Comprehensive Annotation and Functional Exploration of MicroRNAs in Lettuce
Source: Front Plant Sci. 2021 Dec 24;12:781836. doi: 10.3389/fpls.2021.781836 (PMC8739914; doi:10.3389/fpls.2021.781836)
Supplement: Supplementary file 3 [file Data_Sheet_9.ZIP › GSM2230753.plot/Lsa-miR398b_Lsat_1_v5_gn_7_49821.1_1490_TPlot.pdf]

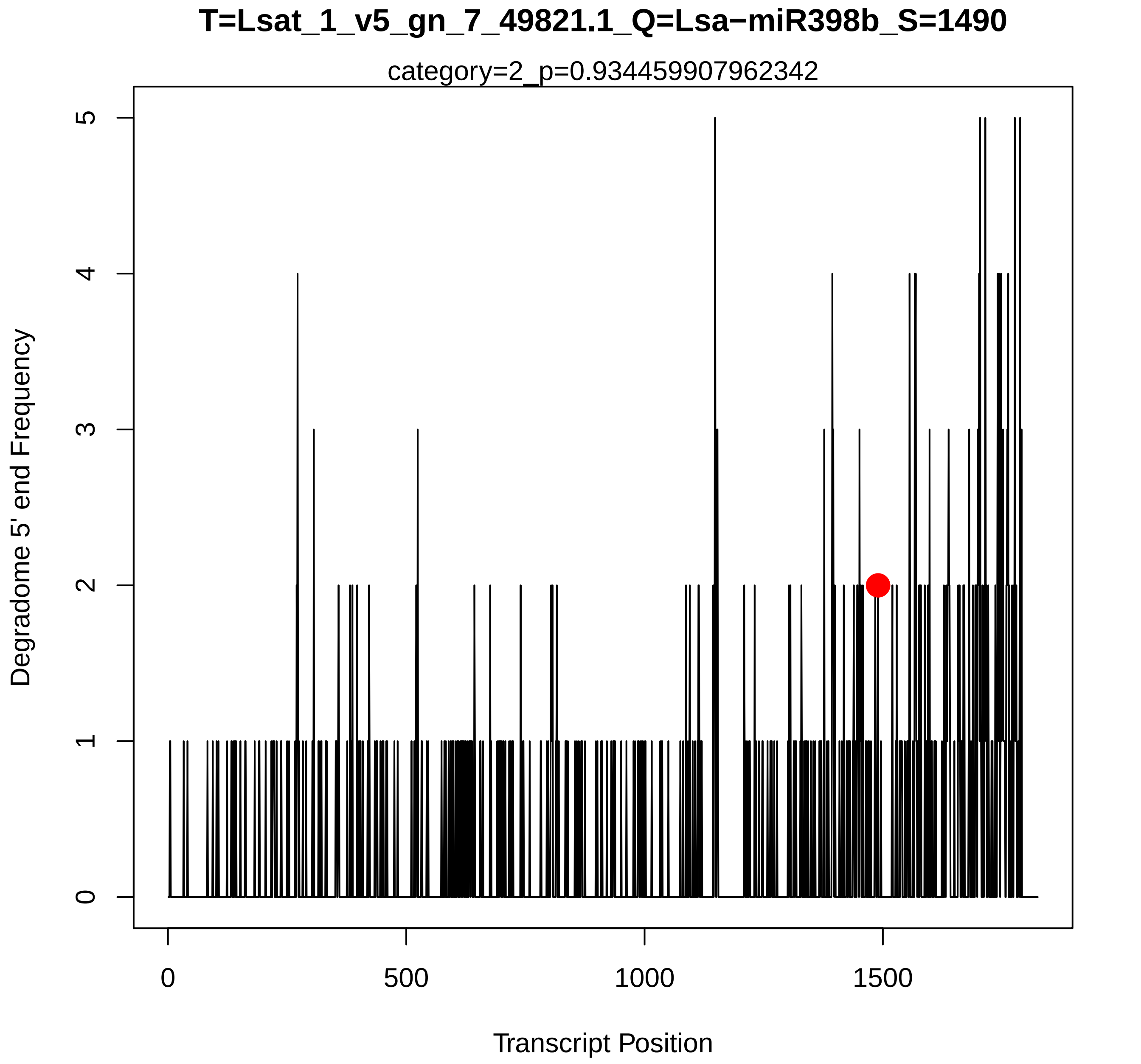

T=Lsat_1_v5_gn_7_49821.1_Q=Lsa−miR398b_S=1490
category=2_p=0.934459907962342
Degradome 5' end Frequency
0
1
2
3
4
5
0
500
1000
1500
Transcript Position
